# Evidence for widespread translation of 5′ untranslated regions

**DOI:** 10.1093/nar/gkae571

**Published:** 2024-07-02

**Authors:** Jose Manuel Rodriguez, Federico Abascal, Daniel Cerdán-Vélez, Laura Martínez Gómez, Jesús Vázquez, Michael L Tress

**Affiliations:** Cardiovascular Proteomics Laboratory, Centro Nacional de Investigaciones Cardiovasculares Carlos III (CNIC), 28029 Madrid, Spain; CIBER de Enfermedades Cardiovasculares (CIBERCV), 28029 Madrid, Spain; Somatic Evolution Group, Wellcome Sanger Institute, Wellcome Genome Campus, Hinxton, Cambridgeshire CB10 1SA. UK; Bioinformatics Unit, Spanish National Cancer Research Centre (CNIO), 28029 Madrid, Spain; Bioinformatics Unit, Spanish National Cancer Research Centre (CNIO), 28029 Madrid, Spain; Cardiovascular Proteomics Laboratory, Centro Nacional de Investigaciones Cardiovasculares Carlos III (CNIC), 28029 Madrid, Spain; CIBER de Enfermedades Cardiovasculares (CIBERCV), 28029 Madrid, Spain; Bioinformatics Unit, Spanish National Cancer Research Centre (CNIO), 28029 Madrid, Spain

## Abstract

Ribosome profiling experiments support the translation of a range of novel human open reading frames. By contrast, most peptides from large-scale proteomics experiments derive from just one source, 5′ untranslated regions. Across the human genome we find evidence for 192 translated upstream regions, most of which would produce protein isoforms with extended N-terminal ends. Almost all of these N-terminal extensions are from highly abundant genes, which suggests that the novel regions we detect are just the tip of the iceberg. These upstream regions have characteristics that are not typical of coding exons. Their GC-content is remarkably high, even higher than 5′ regions in other genes, and a large majority have non-canonical start codons. Although some novel upstream regions have cross-species conservation - five have orthologues in invertebrates for example - the reading frames of two thirds are not conserved beyond simians. These non-conserved regions also have no evidence of purifying selection, which suggests that much of this translation is not functional. In addition, non-conserved upstream regions have significantly more peptides in cancer cell lines than would be expected, a strong indication that an aberrant or noisy translation initiation process may play an important role in translation from upstream regions.

## Graphical Abstract

**Graphical Abstract:**
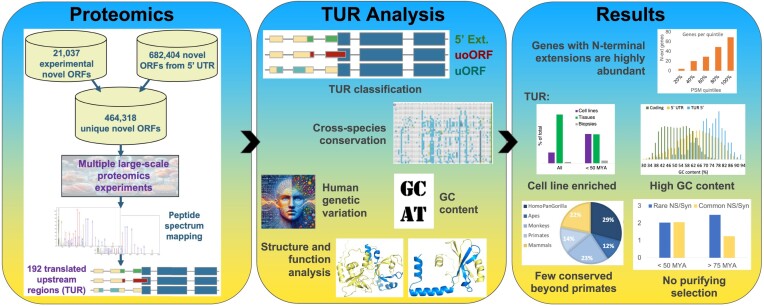

## Introduction

The completion of the heterochromatic regions of the genome by the T2T consortium (1) and the publication of the complete sequence of a Y chromosome (2) means that there is now a comprehensive reference sequence for all 22 human autoosomes and both sex chromosomes. Despite the annotation of a comprehensive human reference assembly, the full set of coding genes in the reference is still some way from being defined.

The T2T consortium has proposed more than a hundred new coding genes (1,2), and several of these genes have already been confirmed to code for proteins (3). In addition, the curators of the two main reference sets, Ensembl/GENCODE (4,5) and RefSeq (6) are continually adding and removing coding genes as new evidence comes to light and existing annotations are revised (7,8). Ensembl/GENCODE, RefSeq and UniProtKB (9). They are now working together via joint projects such as MANE (10) to converge on an agreed set of coding genes.

Early research suggested that humans might have as many as 80 000 genes (11), but since the initial drafts of the human reference genome (12,13), estimates of the number of coding genes in the human reference set have trended stubbornly downwards (14–18). The most recent GENCODE release (v44) annotates 19 396 coding genes.

It has long been suggested that one weakness of the current annotation process is that curators fail to annotate non-canonical small open reading frames (15). One of the problems is that these small ORFs are generally smaller than most coding genes, which makes it harder to find supporting evidence for all but the most obvious cases (19). While several small ORFs have been shown to have functional relevance in recent years (20,21), the avalanche of small ORFs predicted by some has not yet happened (22).

Recently, however, ribosome profiling analyses have found abundant transcript evidence for novel ORFs (23,24). Transcripts captured in ribosome profiling analyses are in the process of being translated. Two recent high profile large-scale analyses have recently provided evidence for thousands of non-canonical ORFs from ribosome profiling experiments (25,26). A consortium has been set up to investigate what role these small ORFs might play in the human genome and how they should be annotated (7).

The two ribosome profiling studies found little reliable support for these non-canonical ORFs in the standard proteomics experiments they carried out. The Chen *et al.* paper (26) found just 10 novel ORFs, for example, and three of these were already annotated as coding. However, they did find considerable evidence for the expression of peptides in human leukocyte antigen proteomics experiments (27).

We reasoned that large-scale proteomics experiments ought to find more evidence for novel ORFs than the standard experiments in the ribosome profiling papers. In an initial analysis, we found evidence for 32 of the 20337 novel sequences predicted from ribosome profiling experiments. Remarkably, 28 of these novel sequences would produce N-terminal extensions of known proteins rather than completely novel proteins, so we concentrated our analysis on the translation of 5′ untranslated regions (UTR).

Translation of 5′ UTR regions is well documented (28–30). Although many of the N-terminal regions detected in the initial studies had considerable cross-species conservation, a more recent analysis of the non-canonical start codons detected in ribosome profiling experiments (31) found that many of the novel upstream regions processed by the ribosome had little evolutionary history.

Here, we searched for evidence of translation of upstream regions by mapping spectra from five large-scale proteomics experiments to 3-frame translations of annotated 5′ exons from the GENCODE reference annotation. We found peptide evidence for translation for 192 upstream start codons. Although some regions had strong conservation evidence, so are clearly protein coding, more than two thirds of these novel translated upstream regions had neither canonical ATG/AUG codons, nor are conserved beyond primates.

## Materials and methods

### Search database for evidence of genome-wide upstream translation

**Figure 1.**
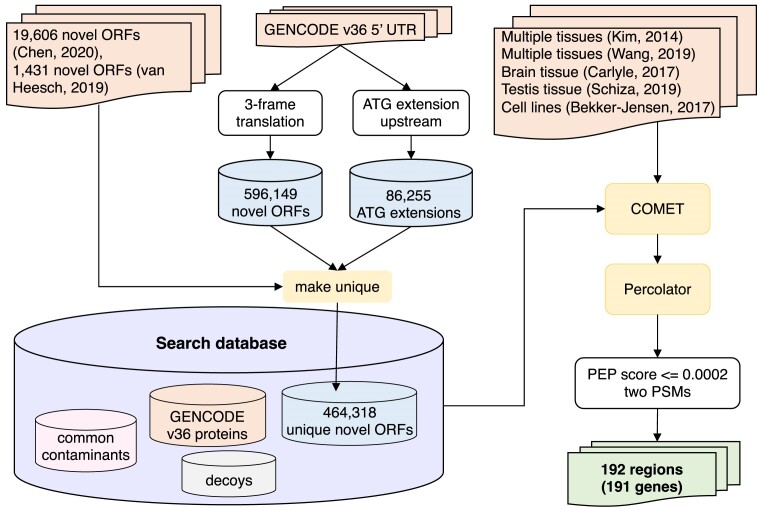
Workflow describing the process of discovering 5′ translated upstream regions.

Our initial analysis is detailed in the Supplementary methods section. For the genome-wide analysis the search database was made up of known and novel protein sequences. We appended the 19606 novel ORFs from the Chen *et al.* analysis (26), which are a mixture of novel isoforms, upstream ORFs and novel ORFs, and the 340 novel lncRNA and 1091 novel upstream ORFs from the van Heesch *et al.* analysis (25) to novel sequences predicted from 3-frame translations of all annotated 5′ untranslated regions (UTRs) in the GENCODE v36 release (4) (Figure 1). Translating the GENCODE v36 5′ UTRs in three frames produced 596149 predicted novel ORFs. Another 86255 predicted novel proteins were generated by extending GENCODE v36 coding sequences upstream from the start codon until we reached a stop codon. These sequences allowed us to detect peptides that mapped to the UTR–coding exon boundary.

A total of 464 318 of the 703 348 predicted coding sequences were unique (Figure 1). To form the search database, these unique sequences were appended to the GENCODE v36 coding gene set from which read-through transcripts had been eliminated (8). The database had both ‘novel’ and known coding sequences, though the proportion of novel sequences was much higher. The database was completed with decoy sequences for both novel and known sequences (decoy sequences were reversed with the tryptic residues maintained, (32) and common contaminants (33).

### Genome-wide proteomics analysis

In this analysis, we searched for translated upstream regions by mapping spectra from five large-scale proteomics experiments to the sequences in the search database (Figure 1). Two of the experiments interrogated a range of normal tissues (29,34), one studied a range of brain tissues (35), another analysed just testis (36), and the final study considered a range of cell lines and biopsy tissues (37).

We chose to analyse tissue-based experiments rather than cell lines, because we were interested in discovering novel regions that were expressed in normal tissues. We added brain and testis experiments to the Kim and Wang analyses because we surmised that there might be more novel regions in these tissues. Finally, we added a large-scale experiment that analysed a range of cell lines, partly as a means of contrast and partly because we know that some proteins are only produced in large quantities during cell division. The combination of experiments allowed us to detect peptides for 14888 coding genes.

Spectra were downloaded from ProteomeXchange (38). Peptide-spectrum matches (PSMs) were generated using COMET (39) with default parameters, including mass tolerance of 10 parts per million, maximum fragment charge of 3 and maximum precursor charge of 4. Only fully tryptic peptides were considered by COMET. We allowed oxidation of methionine as a variable modification. PSMs for peptides between 7 and 40 amino acids in length that were detected by COMET were post-processed with Percolator (40). We used the default parameters in Percolator too, include setting the test and training false discovery rate to 0.01.

Initially we included all PSM with Percolator posterior error probabilities (PEP) values of below 0.001. but we settled on a maximum PEP value of 0.0002 after testing. We decreased the maximum Percolator PEP value because six of the 44 novel peptides mapping to translated upstream regions that had PEP values of between 0.0002 and 0.001 were directly preceded by stop codons, so were almost certainly false positive identifications. This suggests a (minimum) 13.5% false discovery rate (FDR) among novel peptides and shows that a conservative PEP score of 0.001 is too lenient when dealing with novel coding regions (41).

Even at a PEP cut-off of 0.0002, the FDR calculated from the target and decoy novel sequences was 2.83% at the PSM level and 3.65% at the peptide level. Since the final number of peptides used in the analysis of the translated upstream regions was 316, we would expect nine or ten peptides to be false positives. We believe this is a reasonable tradeoff between sensitivity and specificity. The peptide level FDR for novel sequences at the 0.001 PEP cut-off was 11.86%.

We considered only tryptic peptides with up to two missed cleavages in our analysis. As further measure, since we were not able to check all the spectra in this analysis, we required that each novel translated upstream region we identified was supported by at least two PSMs (Figure 1).

### The APPRIS database

APPRIS (42) houses annotations for splice isoforms for a range of species, including human. The database selects a single protein isoform as the cellular representative for each coding gene (the ‘principal’ isoform), based on a series of decisions that make use of cross-species conservation, protein structure and function, and experimental evidence. We have shown that this isoform approximates well to the main cellular isoform (43–45). Non-principal protein isoforms are labelled as ‘alternative’ if they cannot be distinguished from the principal isoform at the initial scoring pass (43), or as ‘minor’ if they are rejected at this stage (42).

### GC-content

We calculated the GC-content for all 5′ UTR, and then used the APPRIS database to separate 5′ UTR from coding genes into 3 types, 5′ UTR of principal transcripts, 5′ UTR of alternative transcripts, and 5′ UTR of minor transcripts. When calculating GC-content for coding genes, we used 5′ UTRs from APPRIS principal transcripts as the reference. Where there was more than one APPRIS principal transcript, we chose the 5′ UTR from the MANE Select transcript (10) when it coincided with the APPRIS principal transcript, and from the longest 5′ UTR when it did not or in those cases where there was no MANE Select transcript. We required all 5′ UTRs to have a minimum of 20 bases. In the case of primate-derived 5′ extensions that were annotated as coding, GC-content was calculated for the coding bases upstream of the principal transcript.

### Human genetic variation

We calculated the non-synonymous to synonymous rates from human germline variants from gnomADg v3.1.2 (46). The effect of the variants was predicted using VEP (47).

The non-synonymous-to-synonymous ratio for both rare and common allele frequencies were calculated for two sets of exons, the 192 translated upstream regions from the 191 genes, and the 262 non-conserved upstream regions annotated as coding in the GENCODE v36 gene set. For the 192 translated upstream regions we could not use the VEP annotations to determine the effect of the variants, as most of these regions were not annotated as coding, and even when they were, they were annotated as coding in a different frame. Instead, we calculated the effect on the predicted protein sequences ourselves.

An allele frequency cut-off of 0.005 was used to separate rare (<0.005) and common (≥0.005) alleles. We also checked how many of these variants would have high impact in the protein, for the sets of common and rare allele frequencies.

### Determining the conservation and start codons of translated upstream regions

We determined the cross-species conservation for translated upstream regions manually, based on the peptide data, the annotated coding exons and the Cactus 100-way and 241-way alignments (48) displayed in the MIT CodAlignView server (https://data.broadinstitute.org/compbio1/cav.php). The translated upstream regions were categorized into one of eight bins of evolutionary origin. We determined which bin a region belonged to depending on whether the reading frame was conserved across other species. The eight bins were: in humans only (i.e. no conservation in any other species), in chimpanzees, in chimpanzee and gorilla, in apes, in monkeys, across all primates, in mammals and in tetrapods (see Supplementary Table S2). For a region to be considered conserved, the orthologous regions in the species that defined the bin had to be largely free of stop codons and/or frameshifts, and the start codon should be conserved. This second rule was not always enforced since the start codon might be in an unannotated exon. In the case of the uORFs and uoORFs only, the stop codon also had to be conserved.

Canonical start codons can be ATG or AUG depending on whether referring to the DNA or RNA sequence. We use ATG exclusively in this analysis because we analysed DNA alignments to determine start codons. As with cross-species conservation, start codons for the upstream regions were also determined from the coding exons, peptide evidence and cross-species alignments. The start codon had to be an ATG codon or at least near-cognate. It also had to be within an annotated 5′ exon and be upstream of all the peptide evidence. Where we found an ATG upstream of the region that produced all the detected peptides, we chose the ATG as the start codon, irrespective of conservation and Kozak sequence strength (49). For upstream regions without an ATG start codon, we looked for possible near-canonical start codons that differed by a single base from an ATG codon (e.g. GTG, CTG, ACG) that could explain all the peptides. Where more than one potential start codon was available, we chose the start codon based on a combination of cross species conservation, Kozak sequence strength (49) and distance from the translated region indicated by the detected peptides. Kozak sequence strength was defined as either strong, defined as position -3 of the Kozak sequence adenine or guanine (49), position +4 guanine, moderate (either position −3 adenine or guanine, or position +4 guanine), or weak (neither position −3 adenine or guanine, nor position +4 guanine).

**Figure 2.**
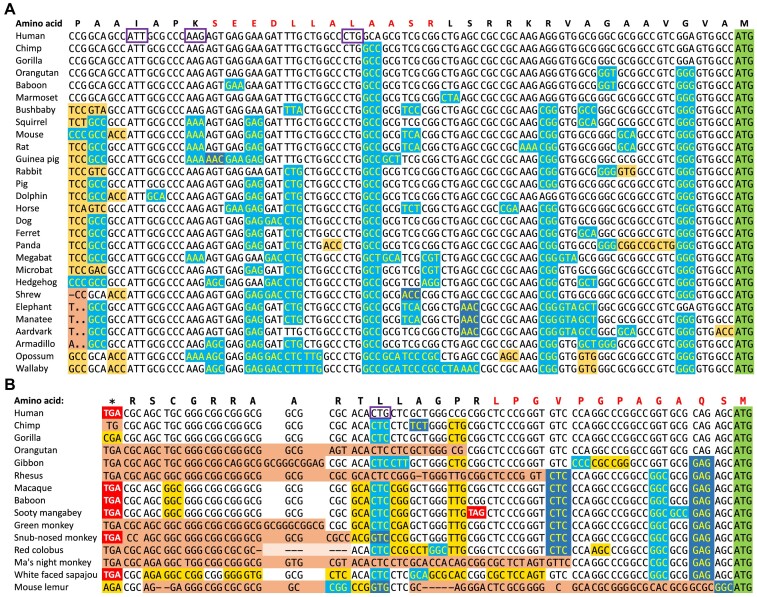
Alignment of orthologous upstream regions for *C1Q4L* and *SLC7A5*. (**A**) The translated upstream region in *C1Q4L*. The orthologous sequences are from eutherian mammals. Alignment and colouring adapted from the CodAlignView server and based on the Cactus 241-way mammalian alignments. Synonymous base changes are shown with a light blue background, non-synonymous changes that would result in conservative amino acid substitutions are shown with a dark blue background, and non-synonymous changes that would produce conservative substitutions are shown with a yellow background. Frameshifts are highlighted in orange. Stop codons are highlighted in red. The annotated downstream ATG is shown with a green background. The detected peptide is shown above the alignment in red font. Potential start codons mentioned in the text are highlighted with a purple box. Synonymous changes greatly outnumber non-synonymous changes suggesting that this region is under strong selective pressure. (**B**) The translated upstream region in *SLC7A5*. Alignment and colouring as for *C1Q4L*. The orthologous sequences are from primates only. Most aligned species have frameshifts or a stop codon. The CTG is only conserved in human.

The process can be illustrated with two examples. The upstream region of *C1QL4* is conserved across eutherian mammalian species in the Cactus alignments (and can trace its origin back to the vertebrate clade). In the upstream region of *C1QL4*, almost all the single base differences are synonymous and there are no frameshifts or stop codons in any other species (Figure 2A). The translated upstream region in *C1QL4* is clearly conserved in eutherian mammal species. Not all cases were as clear as *C1Ql4*.

We determined that the start codon for the *C1QL4* translated upstream region was an ATT codon (Figure 2A). It is upstream of all the peptide evidence and entirely conserved across eutherian mammals. We defined the Kozak sequence as ‘strong’ for the *C1QL4* upstream region. A start codon upstream of the ATT was discounted because there is a frameshift upstream of the ATT in most species, in particular eutherian mammals, so any potential start codon further upstream than the ATT would not be conserved. There are two potential start codons downstream from the ATT. One of these does not fit the peptide evidence (the AAG codon), while the other is not wholly conserved across mammals (CTG codon). In addition, the AAG codon has a weaker Kozak sequence than the ATT codon and in any case selection pressure is evident upstream of the AAG codon.

For the translated upstream region in *SCL7A5*, the selection of start codon is simpler. There is only one possible start codon within the annotated 5′ exon, a CTG that is not conserved beyond humans (Figure 2B). There is no other possible start codon upstream of the CTG, in part because there is a stop codon upstream of the CTG. Most species have frameshifts or a stop codon in the region equivalent to the translated upstream region in human. The frame of the region might be sufficiently conserved among great apes, but the human-specific start codon means that we classified this region as conserved in humans only.

## Results

### Almost all novel sequences supported by proteomics are N-terminal extensions

In an initial analysis of large-scale proteomics experiments (see supplementary results), we found that almost all the ‘novel ORFs’ from the Chen *et al.* (26) and van Heesch *et al.* (25) analyses that had peptide support in these large-scale proteomics analyses were N-terminal extensions of known protein coding genes. We detected peptides for 30 of the 20337 novel ORFs that were not already accepted as coding by GENCODE (4). Remarkably, 28 of these novel ORFs were N-terminal extensions produced from unannotated upstream translation initiation sites (see Supplementary Table S1).

Even when we considered the overlapping ORFs and the bias towards smaller ORFs (less amenable to detection in proteomics analysis) in the novel ORFs in the Chen *et al.* and van Heesch *et al.* analyses, the proportion of N-terminal extensions that we detected was still highly enriched. It was more than double what was expected and clearly statistically significant (Fisher exact test < 0.00001).

### Evidence of translation of 5′ UTR in 191 genes from the human gene set

The initial analysis showed a clear enrichment in isoforms with extended N-termini, so we carried out a genome-wide search for translated upstream regions. Translating the 5′ UTR of GENCODE v36 coding exons into three frames generated a list of 464318 predicted novel sequences. We appended these to the annotated coding sequences in the GENCODE v36 gene set and mapped spectra from five large-scale proteomics experiments to this concatenated search database (see methods for details).

Like many other discovery methods, proteomics is prone to making erroneous identifications, particularly when predicting novel coding sequences and small ORFs (50). We attempted to limit the number of false positives by imposing a particularly strict cut-off in Percolator. Even so, at least three peptides are estimated to be false positive matches, and these are unlikely to be the only spurious identifications.

The peptides that we identified for N-terminal extensions in *LSP1*, *MYL6* and *XPO1* were directly preceded by stop codons. The only possible explanation in all cases is that the match between the peptides and spectra are false positives. At a Percolator PEP value of 0.001, still a conservative value, nine of the detected novel peptides would have been adjacent to stop codons. This highlights the difficulties of controlling for false positives when working with novel coding regions.

**Figure 3.**
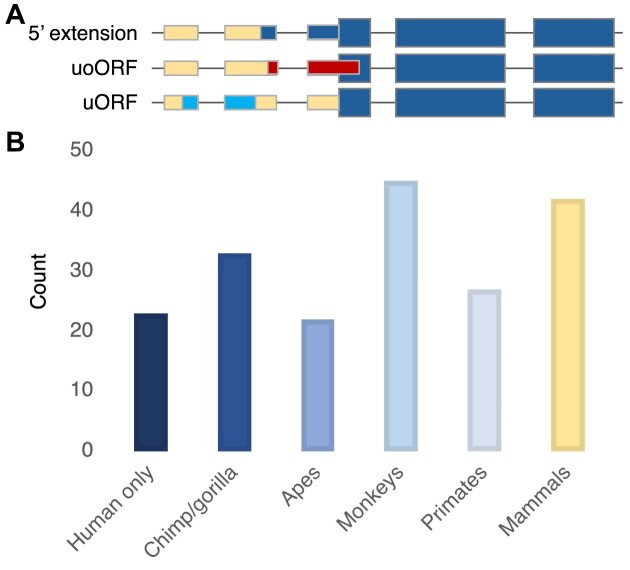
Upstream region types and their cross-species conservation. (**A**) A graphical representation of three types of translated upstream region. Coding exons are represented by thicker boxes, annotated 5′ exons by narrower boxes. The regions of 5′ UTR differently coloured from the background show the differences between the three types of translated regions. 5′ extensions start upstream of the coding exon and run into the coding exon in the same frame, so would generate a protein with a longer N-terminal. uoORFs begin upstream of the coding exon and invade into the coding exon in a different frame. They continue until they reach a stop codon and would produce an entirely different protein. uORFs also begin upstream of the coding exon and would produce a different protein, but they reach a stop codon before the canonical ATG. (**B**) The cross-species conservation of the 192 translated upstream regions separated into six bins. Chimp/gorilla includes all regions only conserved in chimpanzee, gorilla or both. Mammals includes all translated upstream regions that are conserved across mammals at least, though at least sixteen have more ancient origins.

We excluded the upstream regions from *LSP1*, *MYL6* and *XPO1*. The remaining 192 translated upstream regions are listed in Supplementary Table S2. In total, we found 316 peptides for these novel upstream regions (Supplementary Table S3). Most of the upstream regions that we detected peptides for (171) were in-frame 5′ extensions (Figure 3A). But we also found peptides for five upstream ORFs (uORFs) and 16 upstream ORFs that overlapped coding sequence but in a different frame (uoORFs).

Detecting a second peptide for the same novel coding region substantially decreases the probability that the peptide spectrum matches are false positives (51). Even though many of the translated upstream regions are relatively short, 71 of the 192 novel regions (37%) had the support of more than one peptide. Eleven of the 21 uORFs and uoORFs were supported by two or more peptides.

Several other recent studies have identified translation from upstream translation initiation sites (TIS). For example, Kim *et al.* detected peptides for 51 N-terminal exons and 29 uORFs in their analysis of 30 tissues and hematopoietic cells (29), and we coincided with 27 of these in our analysis. Our analysis includes the spectra from the Kim *et al.* analysis, some of the N-terminal extensions we did not confirm may be from transcripts that have been annotated as coding since their study. More recently, Zhu *et al.* (30) found evidence for 53 N-terminal exons in A431 cells and normal tissues, and we confirmed 36, while Na *et al.* (52) detected N-terminally acetylated peptides from 80 genes, and we confirmed 18 of these 5′ extensions. Finally, Fedorova *et al.* (31) detected 447 distinct upstream regions based on ribosome profiling evidence and PhyloCSF conservation (53), and 92 had proteomics support (see Supplementary Table S2). Forty eight of the 447 regions coincided with our translated upstream regions, as well as five of the ‘not translated’ regions. Oddly enough, just two of the N-terminal extensions in our set of translated upstream regions (*FXR2*, *KAT7*) were detected in all four of the analyses.

In addition to the peptides detected for the translated upstream regions, we also detected peptides for a handful of alternative splice variants, pseudogenes, and downstream overlapping ORFs. These are detailed in a section dedicated to novel ORFs in the supplementary results.

### Most translated upstream regions are primate-derived and have near cognate start codons

We located possible ATG or near-canonical start codons for all but 3 of the 192 translated upstream regions. We predicted ATG codons for just 35 regions (17.9%), though nine of 21 (43.9%) uORFs and uoORFs had detectable ATGs. Since we were limited to the annotated 5′ UTR exons, we may have missed the start codons for some translated upstream regions, and it is possible that in some cases there are canonical ATG codons further upstream. This is certainly true in the case of *SERBP1*, where we annotated an ACG start codon. If the annotated UTR were extended somewhat, this translated upstream region would have an ATG codon. The start codons that we did not find are likely to be in unannotated 5′ exons of the three genes.

We traced cross-species conservation within the 192 translated upstream regions. Only 42 (21.9%) were clearly conserved within mammalian species (Figure 3B, Table 1). Almost half of these translated upstream regions (45.2%) were also found in the Fedorova *et al.* analysis. Another 27 (14.1%) had reading frames that were conserved across all primate species, though not in mammals.

**Table 1.** The conservation of translated upstream regions

Last common ancestor	All	5′ ext	uORF/uoORF	ATG
**Human**	23	15	8	5
**Chimpanzees**	14	14	0	3
**Gorillas**	19	16	3	6
**Apes**	22	18	4	3
**Monkeys**	45	41	4	8
**Primates**	27	26	1	1
**Mammals**	38	37	1	6
**Tetrapoda**	4	4	0	3

Last common ancestor is the most distant clade from human in which the reading frame of the 5′ extension, uORF or uoORF is conserved. ATG indicates the numbers of each set that has ATG start codons.

The remaining 123 translated upstream regions (64%) appeared not to be conserved across all primate species. They had multiple premature stops, frameshifts or non-conserved translation start codons in orthologous upstream regions in many or all primate species. Twenty-three did not maintain the coding frame or start codon in any other species (as was the case with *SLC7A5*, for example, Figure 1), and more than a quarter of the translated upstream regions (29.2%) were not conserved beyond gorilla (Table 1). A majority of the 192 translated upstream regions (51%) have neither evidence of conservation beyond monkeys nor canonical ATG start codons and less than 5% have both an ATG start codon and are conserved across mammals.

We looked for evidence of more distant cross-species conservation beyond the Cactus alignments in CodAlignView among the 42 upstream regions that had at least mammalian conservation using the NIH BLAST tool (54). We found that 16 of 42 had evidence for conservation beyond mammals, four in birds and reptiles, five in fish, one in sharks and rays, one in chordates and five in bilaterian species. We found ATG start codons for five of these 16 upstream regions (Table 1).

### Upstream regions with ATG start codons are annotated as coding

Previous studies have also found that the vast majority of unannotated translated upstream regions are translated from near-cognate start codons instead of the cognate ATG codon (30). The fact that just 15% of the 5′ extensions that we found peptides for have an ATG start codon makes sense in the light of the extensive work put into the manual annotation of the human genome (4,6). The reason that we find peptides for so few upstream regions with ATG codons is likely to be that almost all upstream ATGs, even those that do not have the support of cross-species conservation, are already annotated in the human gene set. In fact, we find 262 coding genes with ATG-initiated upstream regions that are annotated as coding, but that are not conserved beyond primates (**see Supplementary results**).

Known near-canonical start codons, such as those in genes *EIF4G2*, and *TEAD1* (55,56), are generally highly conserved. Although a large proportion of the translated upstream regions have little conservation support, we still found a surprising number of regions with well supported non-canonical start codons. The translated upstream region in *VANGL2* has an ATA codon with a strong Kozak sequence which is also almost entirely conserved across all mammalian and tetrapod species, although in some bird species the start codon changes to ATT. It is annotated as an ‘Erroneous initiation Extended N-terminus’ in UniProtKB.

**Figure 4.**
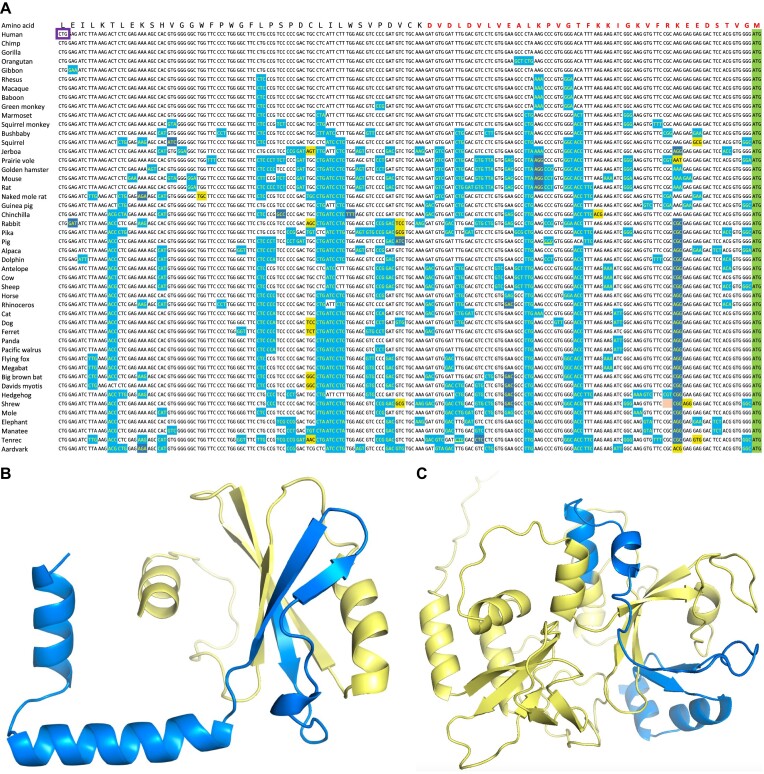
Conserved upstream regions extend functional domains. (**A**) The translated upstream region in *CCDC8*. The orthologous sequences are from eutherian mammals. The alignment and colouring adapted from the CodAlignView server and based on the Cactus 241-way mammalian alignments. Synonymous base changes are shown with a light blue background, non-synonymous changes that would result in conservative amino acid substitutions are shown with a dark blue background, and non-synonymous changes that would produce conservative substitutions are shown with a yellow background. The annotated downstream ATG is shown with a green background. The detected peptide is shown above the alignment in red font. The start codon is highlighted with a purple box. (**B**) The Alphafold (59) model for coiled coil domain containing 8 from Iberian lynx downloaded from UniProt (A0A485NL47) with the novel human N-terminal sequence painted onto the structure. The novel region coded by the translated upstream region (in yellow) completes a PNMA N-terminal RRM-like domain. (**C**) The Alphafold model for Helicase with zinc finger 2 (from gene *HELZ2*) from Pallas’ mastiff bat downloaded from UniProt (A0A7J8HGE4) with the novel human N-terminal sequence painted onto the structure. The novel region coded by the translated upstream region (in yellow) completes a globular structural domain.

Another clear example is *CCDC8*, where the upstream region is composed of 70 amino acid residues, almost entirely conserved across 241 mammals (Figure 4A). Translation is initiated from a conserved CTG codon with a strong Kozak motif. The addition of these 70 residues completes a PNMA N-terminal RRM-like domain (57). The N-terminal region of *CCDC8* has strong similarity to the PNMA family, proteins that have retroviral origins (58). Unlike PNMA genes which are generally brain and testis restricted, *CCDC8* is widely expressed.

Both *VANGL2* and *CCDC8* are now annotated as coding in GENCODE, though the *C1QL4* translated upstream region is not. A less clear example of a potential non-canonical initiation site can be found with the gene *HELZ2*. Here the translated upstream region adds more than 200 amino acid residues that would include two zinc finger motifs and would clearly complete a structural domain (Figure 4B). The upstream region is even conserved in Bilaterian species. We predict that *HELZ2* is initiated from a GTG codon with a strong Kozak sequence. However, although the *HELZ2* translated upstream region is highly conserved, the GTG codon is only conserved among monkeys. In most, but not all, mammalian species the equivalent codon is an ATG. Despite this, it is possible that the true start codon is in a not yet annotated upstream exon.

### Analysis of non-canonical start codons

We predicted near-canonical start codons for 154 translated upstream regions. Three quarters (74.7%) had start codons that differed from the canonical in position 1 of the codon, particularly CTG (70) and GTG (41) codons. There were also 25 start codons that swapped a cytosine for thymine in the second position of the codon (the ACG codon), but the remaining possible non-canonical start codons appeared to be less frequently used (six ATT, four AGG, three AAG, and one ATA). That the CTG codon has most evidence fits with previous findings, since it has been shown to be the most efficient near-canonical start codon (60).

While many of our translated regions overlapped with those of previous studies (30,31,52), there was less agreement than might be expected on the start codons. Among the 48 regions identified both here and in the Fedorova study (31), just 19 had the same start codon. One curious result was that Fedorova *et al.* detected evidence for upstream regions for genes *C1QL3* and *C1QL2*, but not *C1QL4* (Figure 2A). The authors predict CTG and GTG start codons for these two genes, while we predict an ATT start codon for *C1QL4*. Unlike the other two potential start codons, the potential ATT start codon is preserved in all three genes and also in gene *C1QL1*. In all four genes, this ATT start codon is conserved across mammals and it also appears to be conserved in the earliest vertebrates (Supplementary Figure S1). This suggests that all C1QL genes use the same non-canonical upstream TIS. Another family with known near cognate start codons is the transcriptional enhancer family. It seems likely that *TEAD1*, *TEAD3* and *TEAD4* also all use the same non-canonical start codon, a start codon that is conserved in sharks and rays (Supplementary Figure S1).

Only ten of the 20 regions highlighted both in our analysis and the Na *et al.* study (52) had the same start codon. Two genes even had different predicted start codons in our analysis and in those of Fedorova and Na. These were *KCTD3* (CTG, TTG and GTG) and *HDGF* (ATT, TTG and GTG). This illustrates the difficulty in determining the exact start codons when multiple non-canonical start codons are available. Agreement on the predicted start codon was stronger between our analysis and the Zhu *et al.* study (30), though still not perfect. Thirty of the 37 translated upstream regions common to the two studies had the same start codon.

### Translated upstream regions have an elevated GC-content

The mean GC-content of the entire human genome is 41% (12), but GC-content rises to 52.3% among coding exons (61), with the first exon having the average highest GC-content. The GC-content of 5′ UTR is even higher—it is 58.3% over GENCODE v36 5′ UTR. Curiously, functionally relevant coding transcripts have higher GC-content than less functionally relevant transcripts. The GC-content of the 5′ UTRs of transcripts tagged as APPRIS principal (59.8%) or alternative (59.9%) was substantially higher than that of the 5′ UTR of minor transcripts (57.2%). Transcripts tagged as minor by APPRIS make up a large majority of annotated coding transcripts.

**Figure 5.**
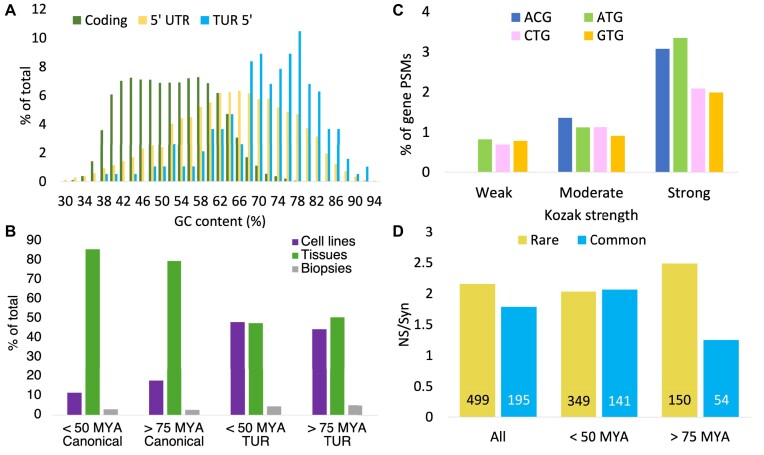
Translated upstream region characteristics. (**A**) Distribution of the GC-content of the exons containing the translated upstream regions (blue) versus principal 5′ UTR (yellow) and principal coding transcripts (green). (**B**) The proportion of PSM in three types of proteomic experiments, tissues, cell lines and biopsies for genes with upstream translations. PSM have been divided into four groups by age of translated upstream regions, either non-conserved (<50 MYA) or conserved at least in Strepsirrhini (>75 MYA), and by whether the peptide mapped to the annotated gene (Canonical) or to the upstream region (TUR). (**C**) Protein expression and Kozak strength. Plots the percentage of the PSMs for genes that map to the N-terminal extensions versus the strength of the Kozak motif for four different start codons (ACG, ATG, CTG and GTG). (**D**) Non-synonymous to synonymous ratios (NS/Syn) for rare and common alleles for all translated upstream regions, for recently evolved translated upstream regions (<50 million years) and for translated upstream regions conserved at least in Strepsirrhini (>75 MYA).

Remarkably, GC-content of the 5′ UTRs from genes with translated of upstream regions, was higher still, 69.4% of the bases in exons housing translated upstream regions were either guanine or cytosine (Figure 5A). This is an exceptionally high GC-content, a third higher than the average for coding exons (52.3%), and more than two thirds higher than the average of the whole genome. This high GC-content may partly explain the bias towards non-canonical start codons. Regions with high GC-content will also have longer ORFs because there will be fewer stop codons.

### Genes with translated upstream regions are highly expressed

We used the total number of peptide-spectrum matches (PSM) detected across the five large-scale analyses as a proxy for protein level expression. Although the total number of PSM detected is partly related to protein length, it also indicates whether a protein product is widely expressed.

Over the five large-scale experiments the mean PSM per gene for the 14 888 genes that we detected peptides for was 425.2. By way of contrast, the 191 genes with evidence of upstream translation had an average of 762.3 PSM per gene. All the difference was due to the genes with 5′ extensions; the 21 genes with detected peptides for uORFs and uoORFs had an average of 411.4 PSM per gene, close to the average for all detected genes.

Genes that produce N-terminally extended isoforms are much more highly expressed than other genes. The 170 genes with 5′ extensions had an average of 808.4 PSM per gene, almost double that of the background. The difference was even more obvious with non-conserved N-terminal extensions. Genes with 5′ extensions that have no evidence of conservation beyond primates had a mean of 866.7 PSM per gene. This is more than double the PSM of background genes, and only a little over 2000 genes had more PSM than this in our analysis.

The fact that we are detecting peptides for N-terminal extensions for the most highly expressed genes suggests two things. Firstly, that the upstream start codons that produce the N-terminal extensions in these genes are not used exclusively and may even be used much less frequently than the canonical start codons. Secondly, it is reasonable to assume that there will also be translation of 5′ extensions in less highly expressed genes, but our proteomics analysis is not capable of detecting it. Proteomics experiments struggle to detect peptides from proteins that have low expression levels (62).

### Tissue specificity of translated upstream regions

The five large-scale experiments that we analysed interrogated a total of 43 tissues, 6 cell lines and 3 patient samples. In our analysis, the vast majority of the PSM that mapped to annotated coding exons came from normal tissues. Just 17% of the detected peptides were from cell lines. The cell line samples were among the experiments with most PSM overall, but they were dwarfed by the PSM from brain and testis (Supplementary Figure S2).

Almost half, 46.3%, of the PSM that we detected for translated upstream regions were detected in the 6 cell lines, more than twice as much as would be expected. This agrees with the results of Zhu *et al.*, who found evidence for more than four times as many N-terminal extensions in A431 cell lines as there were in normal tissues.

Once again, we separated translated upstream regions without cross-species conservation from those that were conserved and found differences between the two sets (Figure 5B). For genes with translated upstream regions that are conserved across all primates the proportion of PSM detected in cell line experiments was 17.7% for peptides that mapped to annotated principal isoforms (close the average for all genes) and 44.4% for peptides that mapped to translated upstream regions. Peptides for translated upstream regions were detected more than twice as often in cell lines. For genes with translated upstream regions that are not conserved across primates, just 11.4% of the PSM from peptides that map to annotated principal isoforms were detected in cell line experiments against 48% of the PSM for peptides that map to the translated upstream regions, Non-conserved translated upstream regions are detected more than 4 times as often in cell lines as the principal isoforms for the same genes.

Evidence for tissue-specific expression of these upstream regions was weak. Where we could measure it, the tissue specificity of the upstream regions followed that of the gene in almost all cases. Tissue specificity is discussed in more detail in the Supplementary results.

### Kozak sequence strength affects translation

We found translation initiation sites for 189 of the translated upstream regions, including all the uORFs and uoORFs. Eighty-one translation initiation sites had strong Kozak sequences, another 94 had moderate Kozak sequences and 14 had weak Kozak sequences. To estimate whether the strength of the Kozak sequence had any effect on the relative translation of the upstream regions, we divided the translation initiation sites of the 168 5′ extensions we found translation initiation sites for by the strength of their Kozak sequence and calculated the percentage of PSMs detected for the translated upstream regions as a percentage of the PSM detected for the genes as a whole.

Those translated upstream regions with weak Kozak sequences produced an average of 6.8 PSM per translated upstream region, which was just 0.78% of the PSM detected for the corresponding genes, while we found 10.4 PSM per region for translated upstream regions with moderate Kozak sequences (1.16% of all PSM from the corresponding genes). Regions with strong Kozak sequences produced more than double the PSMs as the weak Kozak sequences, reaching 15.5 PSM per translated upstream region (2.09% of all PSM detected for the genes). The rule held true for each of the four most common start codons (ACG, ATG, CTG and GTG, see Figure 5C). Stronger Kozak sequences produced more translation of upstream regions than weaker Kozak sequences in our analysis.

### Are translated upstream regions undergoing purifying selection?

Recently derived coding exons have little or no evolutionary track record to support their functional importance. However, human germline variation can shed light on whether a group of exons is currently under selection pressure.

The translated upstream regions that we detected peptides for can be divided into two groups, those that have clear evidence of purifying selection among distantly related species, and those that do not. A total of 42 translated upstream regions are clearly conserved in mammals and so have been under negative selection pressure. Most other translated upstream regions have frameshifts or stop codons in aligned orthologous regions in monkeys, in apes and sometimes even in great apes, so have little evidence of cross-species conservation among other species.

The fact that these upstream regions are translated suggests that these regions may be functionally relevant in humans. We can test for evidence of purifying selection in the translated upstream regions by analysing the numbers of germline variants that would be synonymous and non-synonymous if the region was coding. In the case of the 42 translated upstream regions conserved at least across mammals, we would expect to detect evidence of purifying selection, as long as the translated upstream regions are still functionally important. In the case of those with little evidence of cross-species conservation, we would expect to find evidence of purifying selection only if regions have gained sufficient functional importance.

We analysed non-synonymous to synonymous (NS/Syn) ratios for variants in all 192 translated upstream regions, a total of 694 variants. Over all 192 regions, we found that the NS/Syn ratio was slightly lower for commonly found alleles (1.8) than they were for more rare alleles (2.16), suggesting that at least some of the translated upstream regions are functionally relevant in humans (Figure 5D). This difference was not significant.

We divided the translated upstream regions into two groups, those that were conserved across mammals or at least conserved across all primates (including Strepsirrhini), and those that were not. For the mammal and primate conserved translated upstream regions the NS/Syn ratio for common alleles was half that of rare alleles (1.25 versus 2.49). Although, there were only 54 common variants, too few to detect statistical evidence of purifying selection among the conserved translated upstream regions using dndscv (63), a Fisher exact test found that the distribution of non-synonymous and synonymous variants was marginally significantly different between the common and rare alleles (0.04), confirming that at least some of the conserved translated upstream regions are functionally relevant, as we would expect.

At the same time, translated upstream regions not conserved beyond simians had almost identical NS/Syn ratios for rare (2.03) and common (2.07) alleles. There was no evidence for purifying selection among these translated upstream regions. These regions also had eight potentially high impact frameshifting variants in common alleles. It appears that few, if any, of the non-conserved translated upstream regions have gained functional relevance as proteins.

We also analysed NS/Syn ratios for variants for the 262 non-conserved upstream regions with ATG start codons that are annotated as coding in the GENCODE v36 reference set.

We found that there was no evidence for purifying selection in these upstream regions either (Supplementary Figure S3).

### Annotating translated upstream regions as part of the human gene set

Since we carried out the analysis, several translated upstream regions have been annotated as coding in the human gene set. Ensembl/GENCODE have principally annotated translated upstream regions with conservation support, irrespective of the start codon, for example transcripts in *VANGL2* (conserved in fish species, ATA start codon), *SFPQ* (mammals, GTG), *H1-10* (mammals, CTG), *FXR2* (mammals, GTG), *CYTH2* (reptiles, CTG) and *CCDC8* (fish, CTG) have been added.

Translated upstream regions are also annotated in the RefSeq gene set. Curiously, the two annotation projects seem to have had different annotation criteria because RefSeq have mainly annotated translated upstream regions with ATG start codons, whether these regions have conservation evidence or not. These include *RANGAP1* (conserved in mammals), *SPATA31A1* (monkeys), *NABP2* (chimpanzee), *TPST2* (ATG only conserved apes), *USP10* (ATG only in great apes), and *SHANK3* (a uoORF, and conserved only in human).

**Figure 6.**
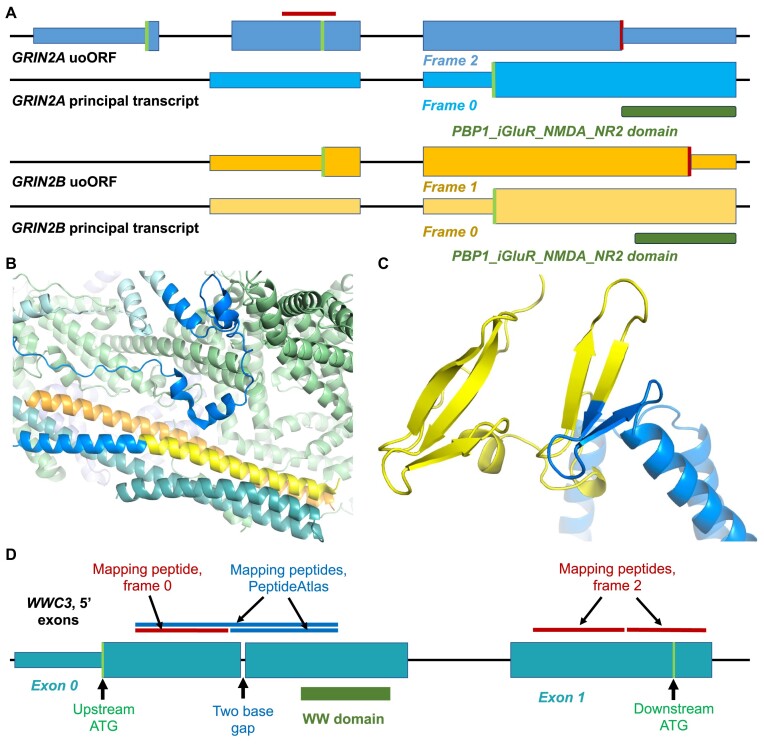
Translated upstream regions in GluN2 genes, in *NHSL1* and in *WWC3*. (**A**) uoORFs in GRIN2A (yellow) and GRIN2B (blue). For each gene there are two sets of exons, the upper set (darker shade) shows the potential coding region of the uoORF, the lower set, the first coding exon of the principal transcript for each gene. Potential coding exons are shown as wide boxes, non-coding exons are more narrow boxes, and introns are black lines. Exons and introns are not to scale. Potential ATG codons are shown as green bars, conserved stop codons as red bars. The frame of each coding ORF is shown (compared to the principal coding exon, which is frame 0). The dark green blocks show where the translation of the LIVBP-like domain (PBP1_iGluR_NMDA_NR2) would start in the principal transcript. The red horizontal line indicates the position of the peptide detected for the *GRIN2A* uoORF. (**B**) The resolved cryo-EM structure of the WAVE regulatory complex (PDB: 7usc, (64)) with the sequence of the *NHSL1* N-terminal extension mapped onto the homologous *WASF1* protein. The *WASF1* protein is in blue and yellow, dark blue where it is homologous to the sequence of the *NHSL1* isoform, yellow where it is similar to the *NHSL1* translated upstream region, and light blue where there was no detectable homology. Homology determined with the HHPRED server (65). The other visible proteins in the complex are the *CYFIP1* protein (light green), the *BRK1* protein (orange) and the *ABI2* protein (teal). (**C**) The Alphafold model for N-terminus of the complete *WWC3* protein downloaded from UniProt (T2C6S4) with the novel human N-terminal sequence painted onto the structure. The novel region coded by the translated upstream region (in yellow) completes a WW domain. (**D**) The upstream exons of *WWC3* (not to scale), with the positions of the upstream and downstream ATGs and the two-base gap marked. Peptides detected for the upstream region mentioned in the text are shown above the exons. Peptides found in our analysis are in red, gap-spanning peptides found in PeptideAtlas in blue.

The *GRIN2A* uoORF is the only translated uoORF that is conserved in mammals. Not only is the reading frame conserved, but there is also evidence of cross species purifying selection. The uoORF has two potential conserved ATG start codons. Both with medium strength Kozak motifs that if translated would produce polypeptides of 57 or 109 amino acids. Since the peptide we detect for the *GRIN2A* uoORF overlaps the downstream ATG, the peptide supports only the upstream ATG (Figure 6A).

*GRIN2A* produces glutamate-binding subunits for the NMDA receptor/ion channel involved in neurite development, synapse formation and synaptic plasticity (66). NMDA receptors are tetramers made up of two GluN1 monomers and two regulatory GluN2 subunits. There are four GluN2 genes, *GRIN2A*, *GRIN2B*, *GRIN2C* and *GRIN2D*, and the two GluN2 subunits in the NMDA receptor can be a combination of any two of the four GluN2 proteins (66). Variants in GluN2 genes are implicated in a range of developmental disorders (67).

The precise combination of GluN2 regulatory subunits within NMDA receptors depends on cell type and developmental stage. GluN2B and GluN2D subunits are most common during early development, while GluN2A and GluN2C production increases after birth. This shift from GluN2B to GluN2A occurs at different time frames in different tissues (68). Curiously, *GRIN2B* also has a conserved uoORF that overlaps the canonical ATG (Figure 5A). Since both genes have conserved uoORFs, it may be that the uoORFs are involved in the regulation of expression of GluN2 subunits.

If translated the *GRIN2B* uoORF would be 80 amino acids residues in length. Despite the coincidence between the two uoORFs, there is no obvious homology. The *GRIN2B* uoORF is in a different frame to the *GRIN2A* uoORF (relative to the coding frame), and the *GRIN2B* uoORF overlaps the LIVBP-like domain, while the *GRIN2A* uoORF does not (Figure 5A).

Unlike the *GRIN2A* uoORF, the *GRIN2B* uoORF appears not to be under purifying selection. Many single nucleotide differences in *GRIN2B* uoORF orthologues are non-synonymous even though the ORF is conserved. For example, 15 of the 20 single nucleotide differences between the *GRIN2B* uoORF and the mouse orthologue would produce radical amino acid changes according to Codalignview, and just one would be synonymous.

The upstream region detected in *NHSL1* (Nance-Horan Syndrome-like 1) is annotated in RefSeq, but not yet in Ensembl/GENCODE. It would extend the N-terminus of an alternative *NHSL1* isoform. The novel upstream region is conserved in orthologues across the bilaterian clade and the ATG and the dozen amino acids that follow are remarkably conserved in species as distant to humans as sea cucumber, molluscs, beetles and spiders. Peptides for the *NHSL1* N-terminal region have been reported previously (69).

Both the novel extended alternative N-terminus and the N-terminus of the principal isoform of *NHSL1*, ENST00000427025, are homologous to the N-terminal coiled coil from *WASF1* (Wiskott-Aldrich syndrome protein family member 1). The *WASF1* protein is part of the Arp2/3-regulating WAVE complex (see Figure 6B) and *NHSL1* has been shown to negatively regulate WAVE-Arp2/3 activity and inhibit cell migration (70). This shared homology shows that the two alternatively spliced N-termini arose by tandem exon duplication (71). Tandem duplicated exon substitution events are mostly ancient (the *NHSL1* duplication can be traced back to the Chondrichthyes clade) and likely to have played important roles in the evolution of tissues and organs (72).

The *NHSL1* protein without the N-terminal interacts with one the members of the WAVE complex, *ABI1*, via the *ABI1* SH3 domain (70), but the homology of the *NHSL1* N-terminals to the N-terminus of *WASF1* suggests that *NHSL1*-mediated WAVE complex inhibition may also be facilitated through the interaction with the N-terminal coiled coil domains of the ABI interactor proteins (Figure 6B).

Two 5′ extensions with both mammalian conservation and ATG start codons (*MROH8* and *WWC3*) have been annotated by both Ensembl/GENCODE and RefSeq. However, both extensions require a change of frame to incorporate both the ATG and peptides. For example, the 5′ extension in *WWC3* involves two exons and contains a WW domain that gives the gene its name and that is highly conserved even across the protostomia clade (Figure 6C). The WW domain is important for interaction with *DVL2* and *LATS1* and has an important role in promoting Hippo signalling (73). The upstream ATG that precedes the translated upstream region certainly seems to have been the ancestral start codon, but a frame-shifting deletion in the first exon specific to humans means that much of the region (including the WW domain) cannot be translated in frame, putting the coding sequence in doubt (Figure 6D). We find peptides upstream of the frame shift and peptides downstream of the frameshift, but if the reference sequence is to be believed, these peptides must have been produced from different frames. There are also peptides in the PeptideAtlas database (74) that cross the indel that causes the frameshift (Figure 6D), so there is considerable evidence that much of the population produces a protein that is not affected by the frameshift.

At present both RefSeq and Ensembl/GENCODE have made a compromise by annotating a two-base deletion in the middle of the upstream exon to account for the frameshift (the PeptideAtlas peptides suggests that there is really a single base insertion). There is an indel variant that would restore the frame (rs1338690519), but it is annotated as being present in 1% or less of the population. Unless there is human-specific ribosomal frameshifting for this exon (unlikely), a different explanation will have to be found to explain all the upstream peptides in *WWC3*.

## Discussion

We detected evidence for translation from 192 distinct 5′ untranslated regions (5′ UTR) regions in large-scale proteomics experiments. There was considerably more peptide evidence for the translation of 5′ UTR than for any other type of predicted novel sequence. Most of these translated regions have stop codons or frameshifts across mammalian species and most would initiate from non-canonical start codons. The abundance of evidence for upstream translation (29–31,52), the use of non-canonical start codons (28,30,31,52) and the lack of cross-species conservation (31) have all previously been reported.

Almost 90% of the translated upstream regions were in-frame with the downstream coding sequence so would produce proteins with extended N-terminal regions. The mean protein expression of genes with these extended N-terminal regions was twice that of the other genes which we detected peptides for. This suggests that the translated upstream regions we detect are only the tip of the iceberg. It would not be a surprise to find that thousands of genes produce can proteins with extended upstream regions.

Given the apparent high frequency of upstream translation, it is important to know whether it has any adaptive benefit or whether it is neutral in nature (75,76). Excess upstream translation may have costs for the cell (76) including wasting cellular resources, attenuating gene function even if the protein product is not toxic. It is known that ATG codons are reduced in number upstream of canonical ATG codons (77,78), so upstream translation is selected against in some genes at least.

The novel upstream regions that we detected peptides for have many features that would not normally be considered indicative of coding regions. More than three-quarters are likely to initiate in non-canonical translation initiation sites, for example. Although most of the non-canonical start codons were not conserved beyond primates, we find non-canonical start codons for 33 translated upstream regions that are conserved in mammals. Non-AUG start codons have been shown to generate proteins in a handful of genes, for example *EIF4G2* (56), which has a role in leaky scanning (79), and *TEAD1* (55), but our results suggest that non-canonical start codons may be more commonly used than thought.

Some of the upstream regions can be traced back to the mammalian lineage and five have equivalent regions in Bilateria. In a number of cases, the upstream regions would complete missing structural and functional domains. This group of sequences appears to be under purifying selection (though we lack sufficient variants to confirm this). These are coding regions that were missed by gene annotators until now.

However, most translated upstream regions have little evidence of cross-species conservation. Equivalent regions across multiple primate species have stop codons or frameshifts that would disrupt them. More than a quarter have no evidence of coding conservation beyond great apes. These non-conserved upstream regions have remarkably high GC-content, allowing longer potential coding regions without stop codons and promoting the generation of near-cognate translation initiation sites. Among these upstream regions there is no evidence of purifying selection at all, which suggests that few, if any, of these regions are under functional constraint. Genes with these regions are among the most highly expressed at the protein level, and it is noticeable that the peptides that we detect are disproportionately enriched in cancer cell line experiments (48% versus a background of 11.4%). Given that translation is known to be dysregulated in cancer cells (27,80,81), it indicates that at least a certain proportion of these peptides may be the result of an aberrant translation process.

Although some translation from these non-conserved upstream regions might be explained by aberrant translation, most of the PSM that we detected for these regions came from normal tissues. Since our results suggest that most non-conserved translated upstream regions are unlikely to have adaptive benefits, the most parsimonious explanation for their translation to protein is that translation is the result of an inefficient translation initiation step.

As part of the canonical translation process, the ribosome binds upstream of the start codon and then scans the mRNA for possible start codons (82,83). If the recognition of the start codon is not efficient, we would expect there to be low-level translation from upstream regions. In proteomics experiments we would expect to detect this low-level translation only in highly expressed genes. This appears to be exactly what is happening.

Low-level translation of non-coding regions will be permitted if the cost to the cell is minimal. Only the more deleterious errors are selected against because the cost of preventing all erroneous translations is high. However, the fact that these regions are translated means that some upstream regions may become useful components of the cell with time. The patterns of human germline variation that we find suggest that some of the translated upstream regions that are conserved in mammals, or at least across all primates, may have gained cellular roles in this way.

## Supplementary Material

gkae571_Supplemental_Files

## Data Availability

There are no new data associated with this article. The datasets were derived from sources in the public domain: the AlphaFold models from the EBi-AlphaFold collaboration (https://alphafold.ebi.ac.uk/), the UniProtKB data from UniProtKB database (https://appris.bioinfo.cnio.es/), the APPRIS annotations from (https://appris.bioinfo.cnio.es/), the human gene set annotations are from GENCODE (https://www.gencodegenes.org/human/), the Cactus alignments and Codalignview annotations from the MIT (https://data.broadinstitute.org/compbio1/cav.php) and the proteomics datasets from ProteomeXchange (https://www.proteomexchange.org/).
