# Supplementary material for: Evidence for widespread translation of 5′ untranslated regions
Source: Nucleic Acids Res. 2024 Jul 2;52(14):8112–26. doi: 10.1093/nar/gkae571 (PMC11317171; doi:10.1093/nar/gkae571)
Supplement: gkae571_Supplemental_Files [file gkae571_supplemental_files.zip › TUR_supplementary.docx]

**Supplementary methods**

**Initial search database**

The initial search database was made up of known and novel protein sequences. ​​We appended the 19,606 novel ORFs from the Chen *et al*. analysis [**Chen *et al*., 2020**], which are a mixture of novel isoforms, upstream ORFs and novel ORFs, and the 340 novel lncRNA and 1,091 novel upstream ORFs from the van Heesch *et al*. analysis [**van Heesch *et al*., 2019**] to the GENCODE v33 human gene set [**Frankish *et al*., 2023**] from which read-through transcripts had been eliminated (**Abascal *et al*., 2018**). There were a total of 20,337 distinct novel sequences from the two ribosome profiling analyses because a number of sequences appeared in both analyses.

Combining these novel sequences with the GENCODE v33 human gene set added just 14,543 unique putative protein sequences because a total of 138 isoforms claimed as novel are annotated as coding in GENCODE v33, and another 5,656 novel isoforms are truncated versions of annotated coding sequences - that is their sequence is identical to a GENCODE v33 isoform, but shorter. These “novel” isoforms usually have a different start codon and cannot be reliably distinguished in shotgun proteomics experiments. The search database was completed with decoy sequences for both novel and known sequences (decoy sequences were reversed with the tryptic residues maintained [**Wright and Choudhary, 2016]** and common contaminants [**Mellacheruvu *et al*., 2013**].

**Initial proteomics analysis**

In the initial proteomics analysis, we matched GENCODE v33 and the 14,543 novel sequences to spectra from just two large-scale tissue-based proteomics experiments [**Kim *et* *al.,* 2014; Wang *et al.,* 2019**]. Spectra were downloaded from ProteomeXchange (**Deutsch *et al*., 2017**). Peptide-spectrum matches (PSMs) were generated using COMET [**Eng *et al*., 2013**] with default parameters, including mass tolerance of 10 parts per million, maximum fragment charge of 3 and maximum precursor charge of 4. Only fully tryptic peptides were considered by COMET. We allowed oxidation of methionine as a variable modification.

The PSMs detected by COMET were post-processed with Percolator [**The *et al*., 2016**]. We set the maximum Percolator posterior error probabilities (PEP) value to a conservative 0.001. We allowed sequences to be validated by a single peptide-spectrum match from a single peptide since we were going to inspect the PSMs. Validated peptides were allowed two missed cleavages. We calculated a separate false discovery rate for the novel PSM using just the novel peptides and their decoys.

**Supplementary results**

**Initial proteomics analysis of novel “ORFs” finds almost all are N-terminal extensions**

We detected at least one peptide for 13,407 GENCODE v33 genes in the two large-scale tissue-based proteomics experiments. In addition, we also detected 53 peptides that mapped 32 “novel ORFs” from the Chen *et al*. and van Heesch *et al*. analyses. Of these novel sequences, two were already part of the GENCODE annotation pipeline because they had been found in previous analyses but were not yet annotated in v33. *ERVH48-1* (suppresyn) has been annotated as coding in UniProtKB for many years, and GENCODE was in the process of reclassifying it as a coding gene (ENSG00000233056). *WASH6P* is almost certainly a pseudogene but was annotated as coding. The peptides detected here most likely are from the new WASH1 coding gene added to chromosome 20 by the T2T-CHM13 assembly [Nurk *et al*., 2022, Cerdán-Veléz and Tress, 2024].

The remaining 30 novel sequences we detected are listed in supplementary table S1. eleven were supported by more than one peptide, and six had the support of 10 or more PSMs. For this set, we manually inspected the spectra for the peptides we detected, and most, but not all, had good matches between the expected and observed fragments. Thirteen of the novel ORFs were also reported by the Kim *et al*. proteomics analysis [Kim *et al.,* 2014].

| **Gene** | **NC ID** | **Peptides** | **Type** |
| --- | --- | --- | --- |
| *ADO^#^* | ADO_64564744_295aa | 1 peptide, 15 PSM | **N-term extension** |
| *ALDH3A2* | ALDH3A2_19552107_544aa | 1 peptide, 10 PSM | **N-term extension** |
| *ATP2A2^#^* | ATP2A2_110719249_1112aa | 2 peptides, 2 PSM | **N-term extension** |
| *C1orf122* | C1orf122_38273505_256aa | 2 peptides, 7 PSM | **N-term extension** |
| *CAVIN2* | SDPR_192711770_465aa | 2 peptides, 3 PSM | **N-term extension** |
| *CCDC8* | CCDC8_46916276_608aa | 2 peptides, 2 PSM | **N-term extension** |
| *CDV3^#^* | CDV3_133292795_289aa | 1 peptide, 1 PSM | **N-term extension** |
| *CYTH2* | CYTH2_48972629_444aa | 1 peptide, 1 PSM | **N-term extension** |
| *DYNC1I1* | DYNC1I1_95402019_653aa | 1 peptide, 1 PSM | **N-term extension** |
| *FXR2^#^* | FXR2_7518068_746aa | 2 peptides, 28 PSM | **N-term extension** |
| *GORASP2* | GORASP2_171785796_470aa | 1 peptide, 1 PSM | **N-term extension** |
| *H1-0* | H1F0_38201308_275aa | 1 peptide, 1 PSM | **N-term extension** |
| *HMGA1^#^* | HMGA1_34204988_146aa | 1 peptide, 1 PSM | **N-term extension** |
| *HNRNPA2B1^#^* | HNRNPA2B1_26240343_390aa | 7 peptides, 137 PSM | **N-term extension** |
| *IQGAP2^#^* | IQGAP2_75699310_1595aa | 3 peptides, 48 PSM | **N-term extension** |
| *KHDRBS3^#^* | KHDRBS3_136469737_470aa | 5 peptides, 8 PSM | **N-term extension** |
| *LINC00969* | LINC00969_3_195663651-195666217 | 1 peptide, 3 PSM | LncRNA/Pseudo |
| *MACROD2* | MACROD2_13976367_439aa | 1 peptide, 1 PSM | **N-term extension** |
| *MOB4^#^* | MOB4_198365112_118aa | 1 peptide, 1 PSM | **N-term extension** |
| *NPLOC4^#^* | NPLOC4_79604012_627aa | 1 peptide, 4 PSM | **N-term extension** |
| *NUDT4* | NUDT4_93772032_202aa | 2 peptides, 26 PSM | **N-term extension** |
| *PPP1R1B^#^* | PPP1R1B_37783399_286aa | 2 peptides, 3 PSM | **N-term extension** |
| *PPP1R2* | PPP1R2_195269874_214aa | 1 peptide, 1 PSM | **N-term extension** |
| *PRKCH* | PRKCH_61788507_787aa | 1 peptide, 2 PSM | **N-term extension** |
| *RINT1* | RINT1_105172723_805aa | 1 peptide, 3 PSM | **N-term extension** |
| *SLC7A2* | SLC7A2_17354693_683aa | 1 peptide, 1 PSM | **N-term extension** |
| *SLC9A3R1^#^* | SLC9A3R1_72744856_401aa | 1 peptide, 1 PSM | **N-term extension** |
| *SORBS2* | SORBS2_186696399_353aa | 1 peptide, 3 PSM | 5’ SS extension |
| *THOP1* | THOP1_2785600_709aa | 1 peptide, 1 PSM | **N-term extension** |
| *TPST2^#^* | TPST2_26940600_417aa | 3 peptides, 5 PSM | **N-term extension** |

**Table S1. The novel ORFs identified in the initial proteomics analysis**

The 30 novel ORFs from the Chen *et al*. and van Heesch *et al*. analyses for which we detected at least one peptide in the Kim *et al*. [Kim *et al.,* 2014] or Wang *et al*. [Wang *et al.,* 2019] large-scale tissue-based proteomics experiments. ORFs that were reported in the Kim *et al*. analysis are marked with a hash symbol. A total of 28 of the 30 ORFs not annotated as coding GENCODE were N-terminal extensions.

What is remarkable is that all but two of the 30 novel sequences are N-terminal extensions produced by the in-frame translation of upstream regions. These translated upstream regions made up just 1,763 of the 20,337 novel ORFs in the search database (8.67%), so N-terminal extensions are clearly enriched in the proteomics analyses. One of the other two novel sequences is actually an alternative splice variant in *SORBS2*. The other is an *SDHA* pseudogene (*LINC00969*) and is highly likely to come from a false positive peptide spectrum match.

Several factors may have contributed to the enrichment of N-terminal extensions in our analysis. Firstly, almost 5,800 of the “novel” ORFs are indistinguishable from GENCODE v33 annotated coding regions (see methods). Secondly, many of the predicted novel ORFs would produce proteins shorter than 50 amino acid residues, and we have found that proteins shorter than 50 amino acids are generally not amenable to detection in standard proteomics procedures [Abascal *et al*., 2018]. In fact, all of the novel sequences for which we detected peptides were from proteins longer than 100 amino acids.

So, in order to determine the significance of finding 28 N-terminal extensions among the proteomics-supported novel ORFs, we excluded novel ORFs that did not differ from those annotated in GENCODE v33 and novel ORFs that were shorter than 100 amino acids. This meant that the search database had just 4,654 detectable novel ORFs, of which 1,701 (36.55%) would produce N-terminal extensions. Even with these numbers, the 87.5% of N-terminal extensions from translated upstream regions that we detected (including the two sequences not yet in GENCODE v33) are still highly enriched. The numbers of N-terminal extensions are clearly significantly higher than would be expected (Fisher exact test < 0.00001).

**Novel sequences detected in the main proteomics analysis**

As well as the peptides for 192 upstream regions, we detected peptides for another 22 novel ORFs in the large-scale analysis. Twelve were downstream overlapping ORFs (doORFs), ORFs that overlapped protein coding genes in a different reading frame, but where the start codon was downstream of the ATG of the principal transcript. Five of these had start codons inside the first coding exon, including the doORF in the gene *CACTIN*, which was supported by six distinct peptides.

The novel doORF in *CACTIN* has an ATG start codon, and both the ATG and the stop codon appeared to be conserved in primates and mostly conserved in mammals. However, this is perhaps not altogether surprising because they both overlap coding regions that are already under selective pressure, albeit in a different frame. It is curious that the doORF in CACTIN overlaps almost perfectly with the region that produces disordered N-terminal leader in the *CACTIN* protein.

None of the other 11 doORFs was supported by more than a single peptide, and none was conserved beyond primates. Apart from the doORFs, there was also support for ten unannotated alternative splice variants (including the one in *SORBS1*), for the ORF1 retroviral protein (LORF_HUMAN), commonly detected in cell lines, and for several pseudogenes. Two of these, *TXLNGY* and *MSL3P1*, may be reclassified as coding by the manual curators in GENCODE.

**Many primate-derived ATG-initiated regions are annotated as coding**

That just one in seven of the 5’ extensions with supporting peptides had canonical ATG start codons suggests that many upstream regions with in-frame ATG codons may already be annotated in coding transcripts in the reference gene set. Most of the 5’ extensions that we found peptides for were not conserved across primates, so many of these annotated ATG-initiated in-frame 5’ extensions might also lack conservation evidence.

We searched for recently evolved N-terminal extensions that are in the same frame as the ATG start codon of the main transcript in the GENCODE v36 reference set. We chose the APPRIS principal transcript as the main transcript. We filtered out isoforms that aligned against at least one non-primate species in the Corsair module of APPRIS [Rodriguez *et al*., 2022]. Corsair analyses BLAST [Altschul *et al*., 2001] alignments of orthologues as part of APPRIS. This is not an exhaustive search, but we still found 262 GENCODE v36 coding genes with recently evolved N-terminal extensions.

Most annotated in-frame extensions have no convincing evidence of conservation in any mammal species. For example, the 68 amino acid N-terminal extension in *IKBKG* is conserved throughout all monkey species but no further. The 67 amino acid N-terminal extension in *CYB561* has no conservation at all since almost all primate species have either stop codons or frameshifts in the equivalent regions. Although there are mammalian orthologues for several of these genes, they are usually erroneous. An ATG present in just human and tree shrew has almost certainly led to the annotation of N-terminal extensions in both species for gene *ZNF668* for example, and the 17 amino acid upstream region in *UNC50* is entirely human specific, yet transcripts with the equivalent upstream region are annotated for several mammalian species including naked mole rat, panda and pacific pocket mouse.

However, the BLAST searches may have missed a small number of N-terminal extensions that evolved in the mammalian clade. One example is in the gene *VKORC1L1* which produces Vitamin K epoxide reductase complex subunit 1-like protein 1, a membrane-bound protein involved in vitamin K metabolism. In *VKORC1L1*, the N-terminal extension composed principally of glycine and alanine residues extends to 47 amino acids in human. Although the upstream ATG in *VKORC1L1* is conserved across primates and many mammals, the glycine-rich section is not even conserved in chimpanzees, which is why we do not detect mammalian homology in Corsair. However, none of the indels in the glycine-rich region led to frameshifts, which suggests that this region may be under selection too.

There is plenty of evidence for peptides for the translated upstream region in *VKORC1L1* [Pozo *et al*., 2022]. Despite this, the *VKORC1L1* N-terminal was the exception rather than the rule. Just 18 of the 262 annotated primate-derived translated in-frame extensions were supported by peptides in our analysis. At first sight it might seem surprising that there is considerably less evidence of translation of 5’ extensions that have ATG start codons than those that have near cognate start codons. However, unlike the 5’ extensions that we found peptides for, the annotated 5’ extension are not highly expressed at the protein level: the mean PSM per gene for the 262 genes with recently evolved annotated 5’ extensions is 425.9, almost identical to the average PSM count of all 14,888 genes we detected peptides for (425.2). We would expect to find more peptide evidence for these regions if their genes were more highly expressed.

These recently evolved 5’ extensions that have been annotated because they have ATG start codons have similar features to the 5’ extensions that we detected peptides for. Both have high GC content and no evidence for selective pressure. GC-content in these regions (61.5%) is higher than the mean GC levels in 5’ UTR, and considerably higher than those of coding exons, though it is not as high as the GC levels in the translated upstream regions we detected peptides for. Analysis of the human variation patterns in the annotated primate-derived translated upstream regions using VEP [McLaren *et al*., 2016] fails to turn up much evidence of selective pressure here either, the ratio of non-synonymous to synonymous (NS/Syn ratio) variants in these exons was 2.08 for rare alleles and 2.04 for common alleles (supplementary figure S3). The percentage of high impact variants (stop lost, stop gained, frameshifts) was higher among the common variants (7.3%) than among the rare variants (5.3%). This increase is likely to be in part because of the low numbers of common alleles (177) and in part to be due the undercounting of rare frameshift variants. Without frameshift variants, the percentages of high impact variants drop to 3.6% for rare alleles, and 4.0% for common alleles. In any case, the percentage of high impact variants is not what would be expected in coding regions.

**Translated upstream regions have limited tissue specificity**

We were curious to know whether any of the peptides that mapped to translated upstream regions were tissue-specific, and if they were, whether their tissue specificity matched that of the corresponding gene. We defined tissue-specific peptides as those that had the majority of their PSM in one tissue, or tissue group, and had at least 3 more PSM in that tissue than in any other tissue (cell lines excluded).

Sixteen translated upstream regions had peptides that fitted our definition. Eight were brain-specific, four testis-specific and four liver-specific. For the comparison against the corresponding gene, we looked at the PSMs from the five experiments and RNA expression evidence in the Human Protein Atlas [Uhlen *et al*., 2016]. We found that the tissue-specificity of 14 of 16 upstream regions concurred with tissue-specificity of the gene, which was reassuring since it confirmed that these upstream peptides were produced in the same tissues as the principal isoform. For example, we found three peptides for the translated upstream region in *KHK* (though only one peptide had enough PSM to qualify for our analysis), and all 6 PSM were from liver samples. *KHK* is liver expressed. *LELP1* is mainly expressed in testis, and all the PSMs we found for the *LELP1* uORF peptide came from testis experiments.

Peptides from the translated upstream regions of 1 of the 16 genes were more enriched in brain tissues compared to the equivalent gene. Expression of the *ALDOA* gene is widespread, yet six of the eight tissue-derived PSM we detected for peptides in the translated upstream region were detected in brain tissues. At the transcript level there is broad expression of *RHOB*, though half the PSM we detected for the *RHOB* protein were from brain tissues. The single peptide that mapped to the translated upstream region in *RHOB* was even more enriched in nervous tissues than the PSM that mapped to the *RHOB* principal isoform. Ten of the 11 PSM we detected for the peptide from the translated upstream region came from brain or spinal cord tissues. A Fisher exact test (0.011) suggests that the peptide from the *RHOB* translated upstream region is significantly more expressed in nervous tissues than would be expected.

**References**

Abascal,F., Juan,D., Jungreis,I., Kellis,M., Martinez,L., Rigau,M., Rodriguez,J.M., Vazquez,J. and Tress,M.L. (2018) Loose ends: almost one in five human genes still have unresolved coding status. Nucleic Acids Res., 46, 7070–7084.

Altschul,S.F., Gish,W., Miller,W., Myers,E.W. and Lipman,D.J. (1990) Basic local alignment search tool. J. Mol. Biol., 215, 403-410.

Cerdán-Vélez,D. and Tress,M.L. (2024) The T2T-CHM13 reference assembly uncovers essential WASH1 and GPRIN2 paralogues. Bioinformatics Advances, 4, vbae029.

Deutsch,E.W., Csordas,A., Sun,Z., Jarnuczak,A., Perez-Riverol,Y., Ternent,T., Campbell,D.S., Bernal-Llinares,M., Okuda,S., Kawano,S. *et al.* (2017) The ProteomeXchange consortium in 2017: supporting the cultural change in proteomics public data deposition. Nucleic Acids Res., 45, D1100-D1106.

Eng,J.K., Jahan,T.A. and Hoopmann,M.R. (2013) Comet: an open-source MS/MS sequence database search tool. Proteomics, 13, 22-24.

Frankish,A., Carbonell-Sala,S., Diekhans,M., Jungreis,I., Loveland,J.E., Mudge,J.M., Sisu,C., Wright,J.C., Arnan,C., Barnes,I. *et al*. (2023) GENCODE: reference annotation for the human and mouse genomes in 2023. Nucleic Acids Res., 51, D942–D949.

Fedorova,A.D., Kiniry,S.J., Andreev,D.E., Mudge,J.M. and Baranov,P.V. (2022) Thousands of human non-AUG extended proteoforms lack evidence of evolutionary selection among mammals. Nat. Commun.,13, 7910.

Kim,M.S., Pinto,S.M., Getnet,D., Nirujogi,R.S., Manda,S.S., Chaerkady,R., Madugundu,A.K., Kelkar,D.S., Isserlin,R., Jain,S. *et al.* (2014) A draft map of the human proteome. Nature, 509, 575–581.

McLaren,W., Gil,L., Hunt,S.E., Riat,H.S, Ritchie,G.R., Thormann,A., Flicek,P. and Cunningham,F. (2016) The Ensembl Variant Effect Predictor. Genome Biol., 17, 122.

Mellacheruvu,D., Wright,Z., Couzens,A.L., Lambert,J.P., St-Denis,N.A., Li,T., Miteva,Y.V., Hauri,S., Sardiu,M.E., Low,T.Y. *et al.* (2013) The CRAPome: a contaminant repository for affinity purification-mass spectrometry data. Nat. Methods, 10, 730–736.

Nurk,S., Koren,S., Rhie,A., Rautiainen,M., Bzikadze,A.V., Mikheenko,A., Vollger,M.R., Altemose,N., Uralsky,L., Gershman,A. *et al.* (2022) The complete sequence of a human genome. Science, 376, 44–53.

Pozo,F., Martinez Gomez,L., Rodriguez,J.M., Vazquez,J. and Tress,M.L. (2022) APPRIS Principal Isoforms and MANE Select Transcripts Define Reference Splice Variants. Bioinformatics, 38, ii89–ii94.

Rodriguez,J.M., Pozo,F., Cerdán-Vélez,D., Di Domenico,T., Vázquez,J. and Tress,M.L. (2022) APPRIS: selecting functionally important isoforms. Nucleic Acids Res., 50, D54–D59.

The,M., MacCoss,M.J., Noble,W.S. and Käll,L. (2016) Fast and Accurate Protein False Discovery Rates on Large-Scale Proteomics Data Sets with Percolator 3.0. J. Am. Soc. Mass. Spectrom., 27, 1719-1727.

Uhlén,M., Fagerberg,L., Hallström,B.M., Lindskog,C., Oksvold,P., Mardinoglu,A., Sivertsson,Å., Kampf,C., Sjöstedt,E., Asplund,A. et al. (2015). Proteomics. Tissue-based map of the human proteome. Science, 347, 1260419.

UniProt Consortium (2023) UniProt: the Universal Protein Knowledgebase in 2023. Nucleic Acids Res., 51, D523–D531.

Wang,D., Eraslan,B., Wieland,T., Hallström,B., Hopf,T., Zolg,D.P., Zecha,J., Asplund,A., Li,L.H., Meng,C. *et al.* (2019) A deep proteome and transcriptome abundance atlas of 29 healthy human tissues. Mol. Syst. Biol., 15, e8503.

Wright,J.C. and Choudhary,J.S. (2016) DecoyPyrat: Fast Non-redundant Hybrid Decoy Sequence Generation for Large Scale Proteomics. J. Proteomics Bioinf., 9, 176–180.

**Supplementary figures**

**
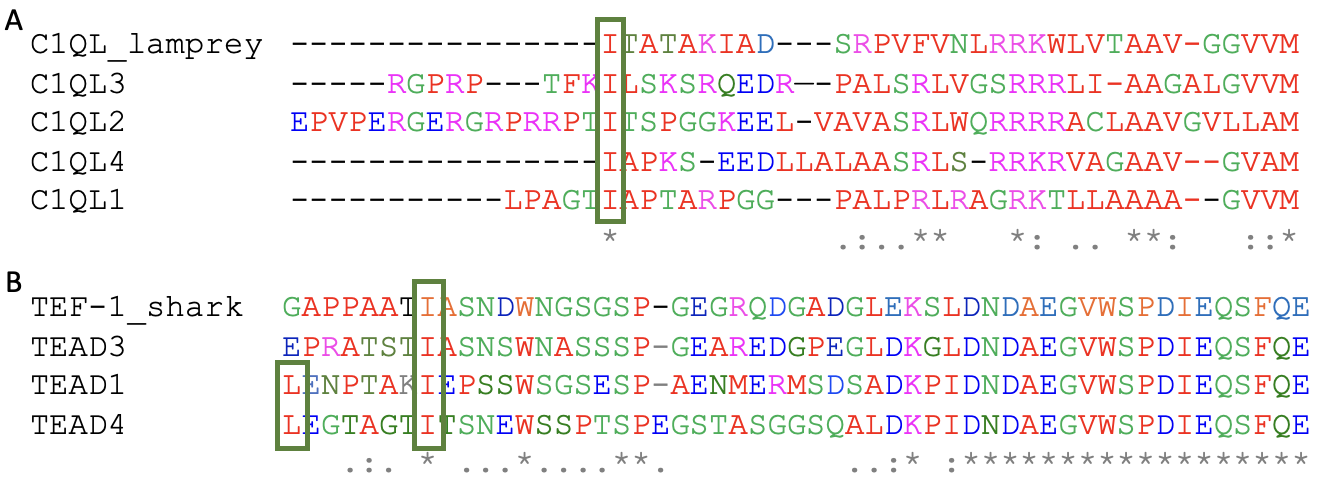
**

**Figure S1. Alignments of upstream regions in the C1QL and TEAD families**

A. Alignment of the four upstream regions detected in C1QL genes in human and the upstream region of the C1QL gene in lamprey. The conserved isoleucine translated from the ATT codon that we believe is the start codon used for the translation of these upstream regions is marked with a green box. The Fedorova *et al* (Fedorova et al. 2022) analysis predicts CTG (leucine) and GTG (valine) start codons form *C1QL3* and *C1QL2*, but these are not conserved in upstream regions in the other genes, B. Alignment of the upstream regions from *TEAD1*, *TEAD3* and *TEAD4*. The conserved isoleucine that would be translated from the ATA start codon in *TEAD3* (and ATT in the other two genes) is marked with a green box. The leucines inside the other green box show the position of the annotated TTG start codon for *TEAD4*. Both the TTG and ATT codons are conserved across mammals, though the ATT codon is completely conserved.


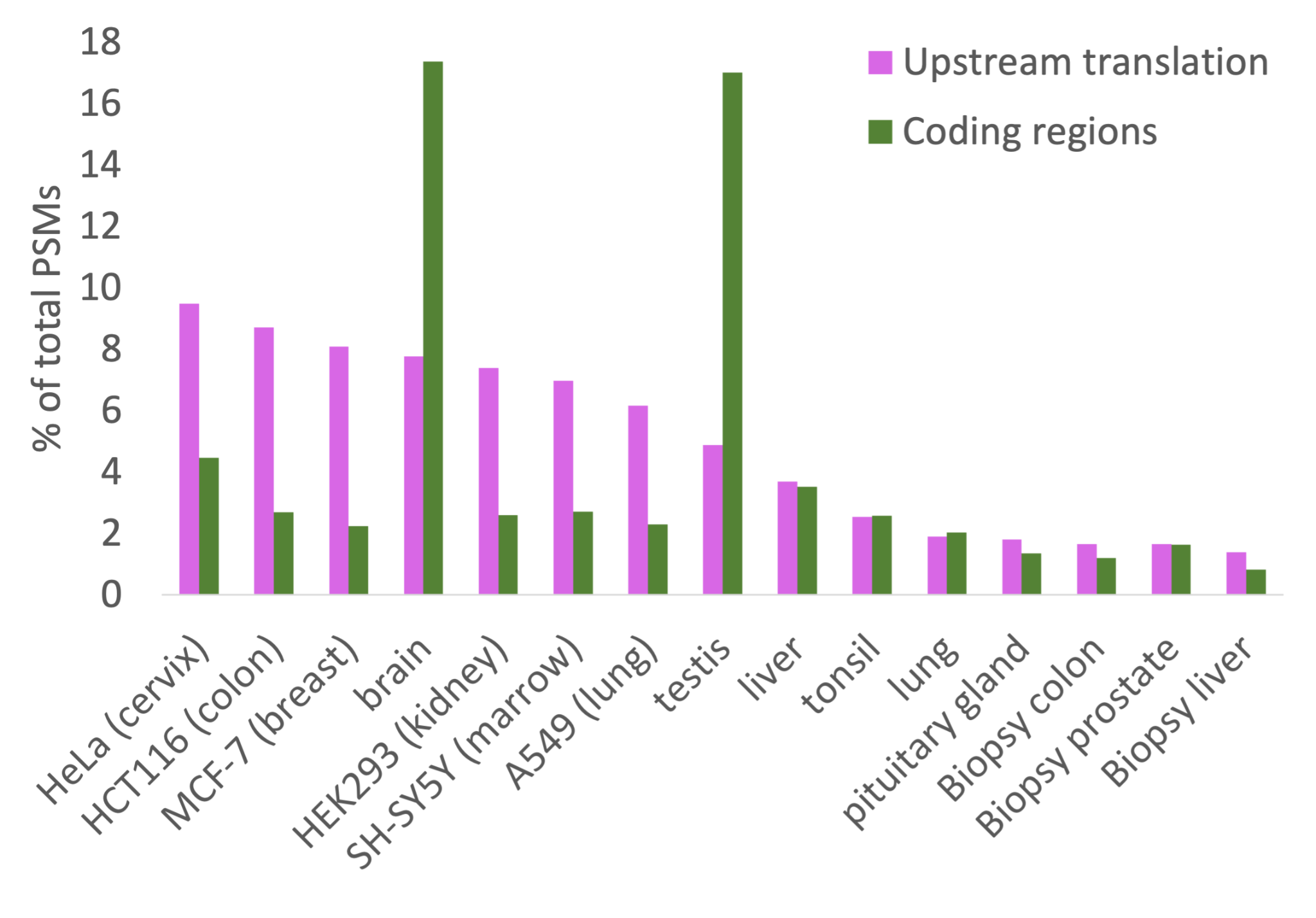


**Figure S2. The samples with the highest percentage of PSMs from upstream translation**

The percentage of all PSM (green) and those PSM that mapped to the translated upstream regions only (pink) for the 15 tissues/cell lines with most PSM that mapped to the translated upstream regions.


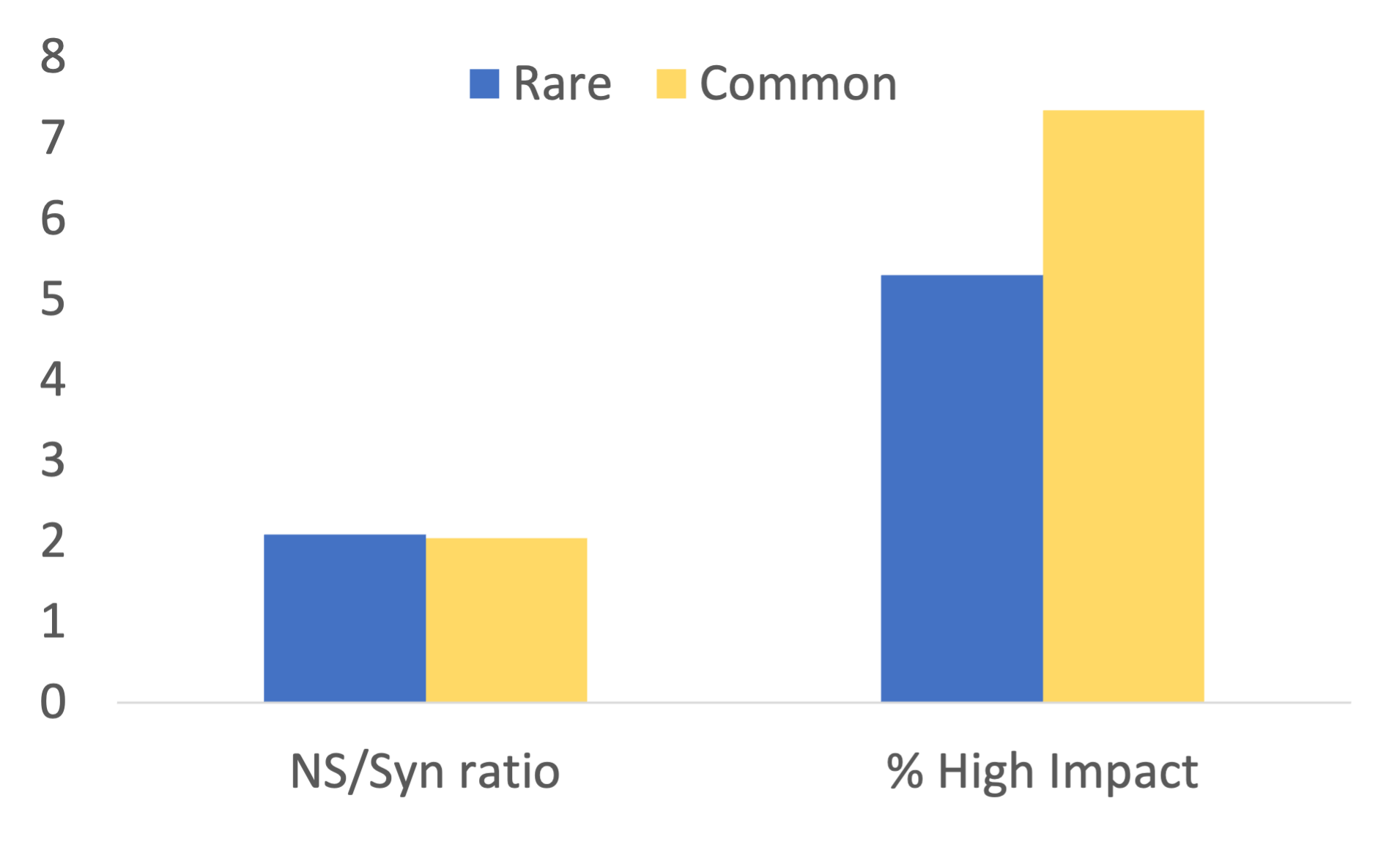


**Figure S3. NS/Syn ratios for annotated not conserved upstream exons**

NS/Syn ratios and the percentage of high impact variants for rare and common alleles among the 262 annotated upstream exons with no evidence of conservation beyond primates.
